# Observational Screening Guidelines and Smartphone Accelerometer Thresholds to Establish the Intensity of Some of the Most Popular Core Stability Exercises

**DOI:** 10.3389/fphys.2021.751569

**Published:** 2021-10-22

**Authors:** Juan R. Heredia-Elvar, Casto Juan-Recio, Amaya Prat-Luri, David Barbado, Francisco J. Vera-Garcia

**Affiliations:** Sports Research Centre, Department of Sport Sciences, Miguel Hernández University of Elche, Alicante, Spain

**Keywords:** trunk exercise, training load, pelvic acceleration, expert rater, postural control

## Abstract

The lack of training load control, mainly exercise intensity, is one of the main limitations of core stability (CS) programs, which makes the training individualization and the analysis of the dose-response relationship difficult. The objectives of this study were to assess the inter-and intra-rater agreement when using new observational screening guidelines to decide if a core stability exercise represents an adequate training intensity level for a given participant. Besides, the relationship between experts' ratings based on these criteria and pelvic accelerations recorded with a smartphone accelerometer was also analyzed. Ten healthy physically active participants with a smartphone accelerometer placed on their pelvis were video-taped while performing a progression of seven variations of the front bridge, back bridge, side bridge and bird-dog exercises. Two expert and four non-expert raters watched the videos and used the observational screening guidelines to decide for each exercise variation if it represented an adequate training intensity level or not. In order to analyze the inter-and intra-rater agreement, several Kappa (κ) statistics were used. Receiver operating characteristic (ROC) curves to explore if the accelerometry allowed to establish pelvic acceleration thresholds representing the minimum level of exercise intensity for CS training. Cut-off acceleration values were calculated balancing sensitivity (Se) and 1-specifity (1-Sp) indexes (i.e., Youden index) or minimizing 1-Sp. The intra-and inter-rater analysis showed a substantial-high level of agreement with a prevalence-adjusted bias-adjusted Kappa > 0.69. The ROC curves showed that the acceleration thresholds for the bridging exercises were very similar, with global cut-off values of 0.35 m/s^2^ (Se = 82%; 1-Sp = 15%) when using the Youden Index and of 0.50 m/s^2^ when minimizing 1-Sp (Se = 31%), whilst the bird-dog exercise showed lower cut-off values (Youden Index: 0.21 m/s^2^, Se = 90%, 1-Sp = 16%; minimizing 1-Sp: 0.32 m/s^2^, Se = 40%). Overall, this study provides observational screening guidelines and smartphone accelerometer thresholds to facilitate the decision-making process when setting the intensity of some of the most popular core stability exercises in young physically active individuals.

## Introduction

Based on the results of previous studies, exercises for improving core stability (CS) have frequently been used as an additional training routine for professional and amateur athletes to improve athletic performance (Sato and Mokha, 2009; Sandrey and Mitzel, 2013; Trecroci et al., 2020) and to prevent and rehabilitate musculoskeletal injuries (Gouttebarge and Zuidema, 2018; Khaiyat and Norris, 2018). In addition, CS exercises have been effective in improving balance, functional performance and preventing the risk of falls in older adults (Granacher et al., 2013) and in reducing pain and disability in chronic low back pain patients (Mueller and Niederer, 2020). Most CS exercises, such as *bridge/plank* and *bird-dog* exercises, consist of maintaining different lying or quadruped postures that challenge the participants' ability to hold a neutral lumbopelvic position (Okubo et al., 2010; Vera-Garcia et al., 2014, 2020; Barbado et al., 2018; El-Gohary et al., 2018). The level of difficulty of these exercises, i.e., the lumbopelvic postural control challenge imposed on the participants, has been related to CS exercise intensity (Barbado et al., 2018) and generally modulated by manipulating different biomechanical constraints (i.e., lever arms, unsupported body mass, number and motion of elevated limbs, base of support, use of labile surfaces, etc.), (Mills et al., 2005; Parkhouse and Ball, 2011; García-Vaquero et al., 2012; Boucher et al., 2016; Vera-Garcia et al., 2020) according to the criteria of the people who select and prescribe the exercises.

Although training intensity is one of the main characteristics of the exercise programs, basic information on how to control and manage the intensity of CS exercises is lacking. For example, there are difficulties in establishing whether a prescribed exercise intensity is appropriate for the participant's level, as well as in determining, after a certain amount of workout sessions, if the given exercises are challenging enough for that participant or if it is necessary to progress toward other more intense exercises. In this sense, although randomized controlled trials on CS training programs usually report that CS exercises are prescribed based on participant's characteristics, the exercise intensity selection and its progression throughout the training program are normally conducted based on the experience and criteria of the professionals who develop the training programs, rather than on objective and quantifiable CS assessments (Cabanas-Valdés et al., 2016; Fox et al., 2016; Prieske et al., 2016; Doganay et al., 2020). Furthermore, the expert criteria used to individualize and modulate the intensity of these CS exercises are not normally specified in these studies (Areeudomwong and Buttagat, 2019; Kim and Yim, 2020). All these limitations hinder the replication of these interventions and do not allow the dose-response characterization of the CS exercise programs (Barbado et al., 2018).

Several biomechanical techniques have been used to objectively quantify the CS exercise intensity and to develop exercise progressions in different populations. In this sense, surface electromyography has traditionally been used to describe the trunk muscle activity intensity during many different CS exercises (García-Vaquero et al., 2012; Vera-Garcia et al., 2014; Calatayud et al., 2017), which supposes an internal index of CS exercise intensity. In addition, post-urographic techniques based on force platforms and smartphone accelerometers have been recently used to assess the participants' difficulty to control trunk posture during CS exercises (Barbado et al., 2018; Guillén-Rogel et al., 2019; Vera-Garcia et al., 2020), which represents an external index of CS exercise intensity. Despite the widespread use of electromyography and force platform post-urography in laboratory settings, their use outside the laboratory is limited due to the cost and complexity of these techniques. On the other hand, considering the low cost, portability, easy use and reliability of smartphone accelerometry (Barbado et al., 2018; Guillén-Rogel et al., 2019), it seems a useful and accessible technique to objectively quantify and control CS exercise intensity in many different contexts (e.g., clinical, athletic and research settings). However, despite the potential of this technique, to the best of our knowledge, no studies have analyzed which acceleration levels represent a sufficient or adequate exercise intensity to induce CS adaptations, nor how changes in the acceleration of CS exercises throughout a training program could be interpreted. Therefore, further research is needed to explore the smartphone accelerometer usefulness to objectively control and manage the intensity of CS exercise programs.

Considering that no literature exists with criteria to help to decide which are the best CS exercise intensity levels for each individual, observational screening guidelines targeting body alignment and postural sway were developed in this study to guide the decision-making process when establishing the intensity level of some of the most popular isometric CS exercises: front bridge, back bridge, side bridge and bird-dog. The main aims of this study were: (i) to analyze the degree of agreement between the evaluations performed by expert and non-expert raters (inter-and intra-rater agreement) using the observational screening guidelines; and (ii) to assess the relationships between the experts' observational assessments and the pelvic sway recorded with a smartphone accelerometer to ultimately try to establish pelvic acceleration thresholds representing the minimum level of exercise intensity for CS training.

## Materials and Methods

### Participants

Ten healthy physically active individuals (males: *n* = 7; age = 26.60 ± 3.13 years; height = 179.14 ± 6.04 cm; mass = 73.00 ± 5.75 kg; females = 3; age = 26.33 ± 1.15 years; height = 167.00 ± 2.65 cm; mass = 65.30 ± 6.75 kg) voluntarily participated in this research. Participants were included in the study if they: (i) did not suffer a disease that contraindicated physical exercise practice (e.g., severe respiratory diseases, hypertension, heart disease, musculoskeletal injuries, etc.); (ii) did not suffer from urinary incontinence; (iii) did not suffer an inguinal hernia; (iv) were under 30 years old; and (v) were not pregnant. Participants were recreationally active, performing 2–5 sessions of 30–120 min of light to vigorous physical activity (jogging, resistance exercises, soccer, gymnastics, cycling, mountain bike, rugby, etc.) per week. None of them participated in a structured CS program at the time of the study, although all of them were familiar with the performance of bridging and bird-dog exercises. At study entry, participants signed an informed consent approved by the University Office for Research Ethics (DPS.FVG.02.14) according to the Declaration of Helsinki.

### Data Collection

The participants completed a single testing session (90 min) in a biomechanics laboratory. They were asked to carry out the testing session barefoot and dressed in short tights and t-shirts. Firstly, the participants filled out a questionnaire about their injury history and their usual physical activity–sports practice. After collecting their anthropometric features (height with the height scale Seca 213®, Germany; mass with the weight scale Tanita BC-601®, Japan), the general characteristics of the CS exercises were explained to the participants and they were encouraged to maintain the spine and pelvis in a neutral position (“as still as possible”) during the exercise execution. Prior to the testing, participants completed a warm-up, which consisted of 10 repetitions of the following exercises: lumbopelvic mobility (i.e., pelvic circles, pelvic anti-versions and retroversions, and cat-camels), twisting crunches, side crunches, trunk extensions and free-weight squats.

**Figure 1:**
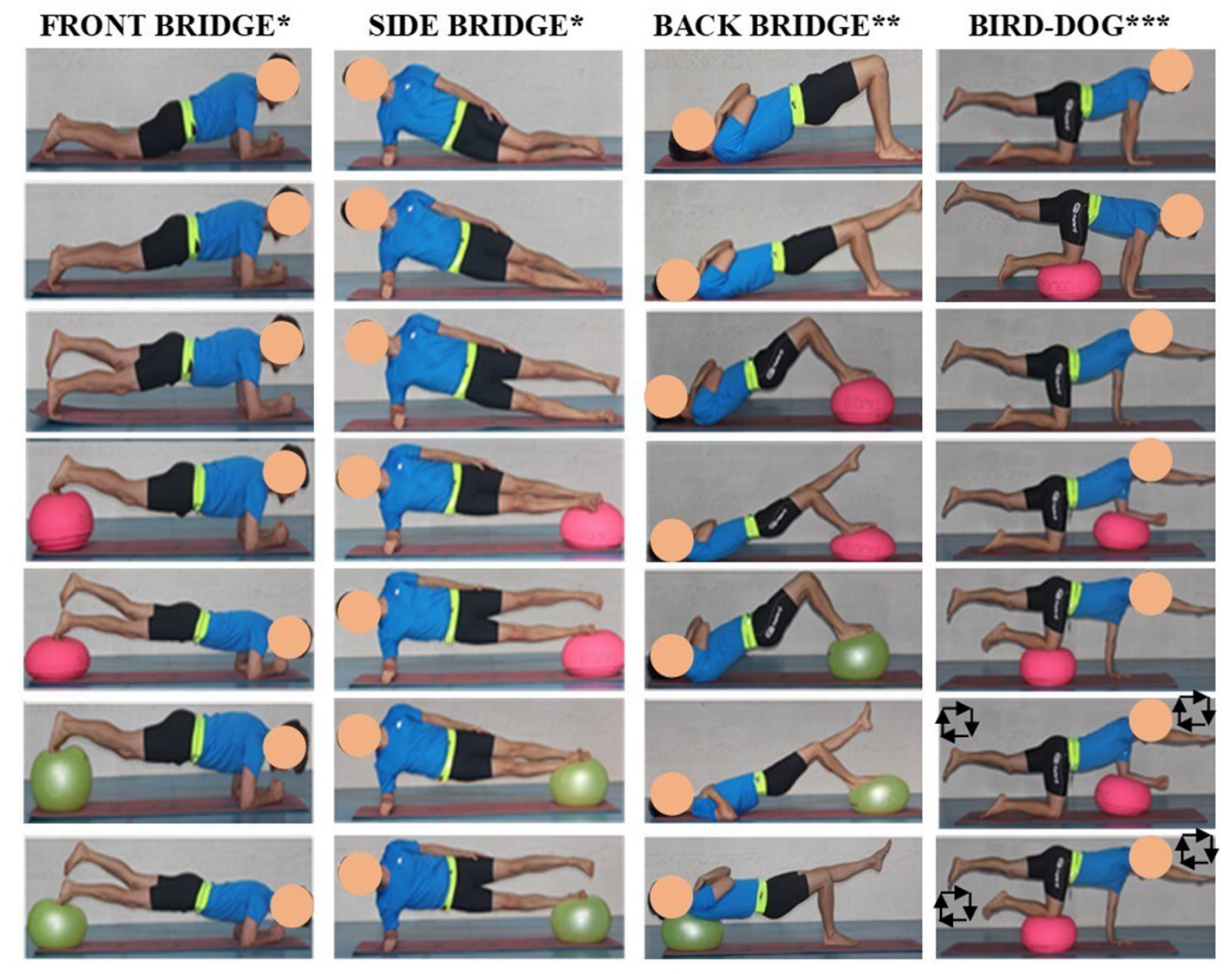
Core stability exercises. ^*^Variations of the *front* and *side bridge* exercises: (1) short front/side bridge; (2) long front/side bridge; (3) front/side bridge with single leg support; (4) front/side bridge with double leg support on a hemisphere ball; (5) front/side bridge with single leg support on a hemisphere ball; (6) front/side bridge with double leg support on a fit ball; (7) front/side bridge with single leg support on a fit ball; ^**^Variations of the *back bridge* exercise: (1) short back bridge; (2) back bridge with single leg support; (3) back bridge with double leg support on a hemisphere ball; (4) back bridge with single leg support on a hemisphere ball; (5) back bridge with double leg support on a fit ball; (6) back bridge with single leg support on a fit ball; (7) back bridge with single leg support and with the upper-back on a fit ball; ^***^Variations of the *bird-dog* exercise: (1) three-point position with an elevated leg; (2) three-point position with an elevated leg and the contralateral knee on a hemisphere ball; (3) classic two-point bird-dog position with elevated contralateral leg and arm; (4) two-point bird-dog position with the forearm on a hemisphere ball; (5) two-point bird-dog position with the knee on a hemisphere ball; (6) two-point bird-dog position with the forearm on a hemisphere ball while drawing squares in the air with the elevated limbs; (7) two-point bird-dog position with the knee on a hemisphere ball while drawing squares in the air with the elevated limbs.

During the testing session, participants performed seven variations of the front bridge, side bridge, back bridge and bird-dog exercises, for a total of 28 variations: (i) for the *front and side bridge* exercises (Figure 1): (1) short bridging, (2) long bridging, (3) bridging with single leg support, (4) bridging with double leg support on a hemisphere ball (54 × 24 cm; Medusa T1, Elksport®, Spain), (5) bridging with single leg support on a hemisphere ball, (6) bridging with double leg support on a fit ball (diameter: 45 cm; Amaya Sport, Spain), and (7) bridging with single leg support on a fit ball; (ii) for the *back bridge* exercise (Figure 1): (1) short bridge, (2) bridging with single leg support, (3) bridging with double leg support on a hemisphere ball, (4) bridging with single leg support on a hemisphere ball, (5) bridging with double leg support on a fit ball, (6) bridging with single leg support on a fit ball, and (7) bridging with single leg support and with the upper-back on a fit ball; and (iii) for the *bird-dog* exercise (Figure 1): (1) three-point position with an elevated leg, (2) three-point position with an elevated leg and the contralateral knee on a hemisphere ball, (3) classic two-point bird-dog position with elevated contralateral leg and arm, (4) two-point bird-dog position with the forearm on a hemisphere ball, (5) two-point bird-dog position with the knee on a hemisphere ball, (6) two-point bird-dog position with the forearm on a hemisphere ball while drawing squares in the air with the elevated limbs, and (7) two-point bird-dog position with the knee on a hemisphere ball while drawing squares in the air with the elevated limbs. The variations of these CS exercises were executed following less-to-more intensity order based on the information provided by a recent post-urographic study on CS exercise progressions (Vera-Garcia et al., 2020). Participants performed all the variations on a single leg with their preferred limb support. In addition, during the bird-dog variations in which participants drew squares in the air, a metronome (60 beats/min) was used to control the pace of the elevated limb motion (participants drew one side of the square every second).

Participants were asked to maintain the appropriate posture for 15 s. This trial duration was selected to avoid participant postural sway changes throughout the exercise because of fatigue. In addition, in order to avoid the large postural oscillations that usually appear at the beginning of the task, the acceleration recording started once the researcher verified that the participant was in the appropriate posture. Each CS exercise variation was performed on a mat (52 × 183 cm; McKinley Trekker M1.3, USA), resting 1 min between trials. Two expert researchers participated in the testing session. One of them controlled the exercise execution and asked the participants to rectify their position when necessary, while the other conducted the post-urography testing.

### Instrumentation and Recording

During the CS exercise performance, pelvic linear accelerations were recorded to evaluate the lumbopelvic postural control challenge imposed on the participants as an index of exercise intensity. Pelvic accelerations were recorded at 200 samples/s from a 3-axis accelerometer (model LIS3DH, STMicroelectronics, Switzerland) embedded in a smartphone (Motorola Moto G, 2013, USA; Chipset Qualcomm MSM 8,226 Snapdragon 400; CPU Quad-core 1.2 GHz Cortex-A7; 1 GB RAM) using a free mobile application (Accelerometer Analyzer, Mobile Tools, Poland). To reduce accelerometer motions caused by muscle contractions, the smartphone was placed between the iliac crest and the great trochanter of the participants' dominant side (the support leg in the single-leg exercises) held in an elastic belt. To control the smartphone remotely and not interfere in the exercise execution, a free remote-control application installed on the smartphone and a laptop (TeamViewer Quick Support, TeamViewer, Germany) was employed.

Two video cameras (Sony Handycam HDR–XR260, Japan and Panasonic FZ200, Japan) were used to record a lateral and an oblique view of the participants performing each exercise variation. The cameras were set up on a tripod at a height of 150 cm above the ground for both views and separated 150 and 200 cm from the exercise mat for the lateral and the oblique view respectively.

### Data Processing

The time series of acceleration data obtained from the accelerometer were filtered using a Butterworth digital filter (4th order, zero-phase lag, low-pass cut-off frequency of 10 Hz). The first second of each trial was discarded, selecting the following 12 s as signal window for the subsequent analyses. Pelvic linear acceleration was analyzed through the mean acceleration, which was calculated as the vector average magnitude in the three axes (Duarte et al., 2014). The acceleration data processing was carried out using a software specifically designed “*ad hoc*” by our research group in Lab View 9.0 environment (v9.0, National Instruments, Austin, Texas, USA).

The recorded videos were edited in a single 15 s long capture that combined the lateral and oblique view using the video editor Camtasia® (version 2020, Tech Smith Corporation, Okemos, Michigan, USA).

### Rating Protocol

Once the videos were edited, they were jointly watched by two CS exercise experts (professors in Biomechanics at bachelor and post-graduate degrees) with more than 10 years of experience in designing, conducting and researching on CS exercise programs. The experts developed a set of observational screening guidelines based on their experience to decide for each exercise variation if it constituted an adequate training intensity level for the participant (*YES-Training level*) or not (*NO-Training level*). For an exercise variation to be rated as YES-Training level, the experts had to consider that *it clearly challenged the participant' CS*, and therefore one of the following criteria were met: (1) the participant showed some difficulty to maintain the head, trunk and limbs aligned and continuously lost and restored the aligned position; (2) the participant showed some difficulty to limit trunk movement (rotation, vibration, tremor, etc.) showing a moderate to high and continuous trunk oscillation around the position. On the other hand, for an exercise variation to be rated as NO-Training level, the experts had to consider that *it did not clearly challenge the participant's ability to maintain the lumbopelvic neutral position*, so both of the following criteria had to be met: (1) the participant maintained the head, trunk and limbs aligned with little or no difficulty; (2) the participant limited trunk movement (rotation, vibration, tremor, etc.) with little or no difficulty while maintaining the body posture. In addition, it was also considered as NO-training level when *the participant was not able to maintain the required position during the exercise variation* (i.e., it was too difficult). Experts were allowed multiple viewings, even pausing or rewinding each exercise progression. They could share their decisions and any discrepancies were discussed until an agreement was reached.

Subsequently, four non-expert raters (Ph.D. students) with 1–3 years of experience in CS training and researching (especially in CS exercises, but not in assessing the intensity of these exercises) attended a training session given by the expert raters. In this session, the non-expert raters watched several video examples of CS exercise progressions while they received feedback from the expert raters on how to decide if the exercise variations showed in the videos represented an adequate challenge/intensity level or not based on the abovementioned criteria. Considering that the lack of rater training standardization may reduce the rating reliability (Eastlack et al., 1991), all the non-expert raters received the same training, and were given an ample opportunity to practice with the exercise variations presented in the videos and ask questions to the expert raters. The non-expert raters were encouraged to rate as YES-Training level those exercise variations in which the *participants clearly showed difficulty maintaining the required posture*, watching the videos of all variations of a given exercise progression before deciding which ones represented an adequate training intensity level and which ones didn't. Besides, the non-expert raters were instructed to rate as YES-Training level all exercise variations that met the established criteria, regardless of the number of variations rated as YES-Training level.

After the training session, the non-expert raters watched the 10 participants' videos and used the experts' criteria to assess the participants' performance and to decide for each exercise variation if it constituted an adequate training intensity level for the participant or not. The same as the expert raters, non-expert raters were allowed multiple viewings, even to pause or rewind the exercise progressions, but they watched the videos and made the decisions alone. To evaluate the intra-rater agreement, the four non-expert raters reassessed the same videos 6 months later to reduce the likelihood that they remembered their previous evaluations.

### Statistical Analysis

In order to analyze the inter-and intra-rater agreement, the standard and multirater Kappa (κ) coefficient, maximum Kappa and observer agreement (Po) and maximum Po were used. To avoid the bias when a higher prevalence of a category existed in the Kappa coefficient, the prevalence-adjusted bias-adjusted Kappa (PABAK) with its confidence limits (CL) was also calculated. The variations for each CS exercise (i.e., front bridge, back bridge, side bridge and bird-dog exercises) were analyzed as individual cases for inter-and intra-rater agreement calculations. Therefore, a total of 70 cases for each CS exercise (10 participants × 7 variations) were included in the analysis. The Kappa and PABAK coefficients were interpreted as: slight agreement (0.0–0.20), fair agreement (0.21–0.40), moderate agreement (0.41–0.60), substantial agreement (0.61–0.80), and almost perfect agreement (0.81–1.00), (Landis and Koch, 1977).

Regarding the acceleration data, the mean and standard deviation of the average pelvic accelerations were calculated for each participant and exercise variation in which she/he was able to maintain the required posture during the whole exercise. Subsequently, the Kolmogorov-Smirnov normality test with the Lilliefors correction was used to verify the normality of the data. Then, to analyze the differences in pelvic acceleration between the exercise variations rated by the experts as YES-Training level and NO-Training level, a one-way ANOVA was performed, being *training level* the between-subject factor (2 levels: YES-Training level and NO-Training level). Besides, to analyze the practical significance of the differences between exercise variations rated as YES-Training level and NO-Training level, the effect size was calculated using the statistical g of Hedge. The effect sizes were interpreted as: large (≥0.8), moderate (<0.8–≥0.5), small (<0.5–≥0.2), and trivial (<0.2), (Cohen, 1992).

Finally, in order to explore if the smartphone accelerometry allowed to classify CS exercise variations as YES-Training level or NO-Training level, a receiver operating characteristic (ROC) curve was calculated for those exercise variations that showed differences between training levels (*YES-Training level* ≠ *NO-Training level*), linking the expert ratings with the average acceleration values obtained by the participants in each CS exercise variation (except those variations in which the participant was not able to maintain the required posture). The area under the ROC curve (AUC) was calculated by comparing it with the non-discrimination value (0.50). For the purposes of our study, acceleration cut-off points were chosen based on two methods. The first method aimed to maximize both sensitivity (Se) and 1-specificity (1-Sp) indexes (i.e., Youden Index) for each exercise variation with the condition that 1-Sp should be <16.7% (i.e., equivalent to one standard deviation). This method was used to reduce the bias caused by the inherent subjectivity of the different raters judging if a CS exercise is challenging or not for the participant. The second method aimed to minimize 1-Sp to remove all the false positives (i.e., exercise variations with acceleration scores over the selected threshold that were categorized as NO-Training level). This more restrictive method was used to ensure that all the exercise variations with acceleration scores over the selected threshold are considered as a sufficient training stimulus based on the experts' criteria, no matter which rater assessed the CS exercise performance. Considering that all bridging exercises (back, side and front bridges) showed similar acceleration scores, the ROC analysis was also applied for all of them together to obtain global pelvic acceleration thresholds.

All statistical analyses were carried out using the Statistical Package for Social Sciences package (SPSS, version 22.0, SPSS Inc., Chicago, IL, USA), establishing a significance level of *p* < 0.05.

## Results

As the inter-and intra-rater agreement results for each CS exercise were very similar, a composite value of all of exercises is presented in Tables 1, 2. Table 1 shows the inter-rater agreement values for the CS exercise variations rated as YES-Training level or NO-Training level based on the screening guidelines. The expert raters rated 61 CS exercise variations as YES-Training level and 219 CS exercise variations as NO-Training level. The observed agreement (Po) was high with values ≥81% in all cases and a value of 0.84 for multiraters^*^experts (Maximum Po = 0.98). The Kappa index ranged between 0.41 and 0.59 among the four non-expert raters and the experts with a multiraters^*^experts' value of 0.53 (Maximum Kappa = 0.93). The PABAK index was ≥0.62 among the four non-expert raters and experts and 0.69 (95% CL = 0.60–0.77) for multiraters^*^experts, which implies a “substantial” agreement.

**Table 1:** Inter-rater agreement for the training level screening criteria between the 4 non-expert raters and the experts.

	**Po**	**Max po**	**Kappa**	**Max kappa**	**PABAK (95% CL)**
**Rater 1/experts**	0.85	0.98	0.56	0.92	0.71 (0.62–0.79)
**Rater 2/experts**	0.86	1.00	0.59	0.99	0.72 (0.64–0.80)
**Rater 3/experts**	0.81	0.96	0.41	0.88	0.62 (0.53–0.71)
**Rater 4/experts**	0.85	0.97	0.58	0.92	0.70 (0.62–0.78)
**Multi-raters/experts**	0.84	0.98	0.53	0.93	0.69 (0.60–0.77)

*Po, Observed agreement; Max, Maximum; CL, Confidence limits; PABAK, Prevalence-adjusted bias-adjusted Kappa*.

**Table 2:** Intra-rater agreement for the training level screening criteria for the four non-expert raters.

	**Po**	**Max po**	**Kappa**	**Max kappa**	**PABAK (95% CL)**
**Rater 1**	0.90	1.00	0.68	1.00	0.80 (0.73–0.87)
**Rater 2**	0.90	0.99	0.67	0.70	0.79 (0.72–0.86)
**Rater 3**	0.90	0.96	0.61	0.85	0.79 (0.72–0.86)
**Rater 4**	0.80	1.00	0.44	0.99	0.59 (0.50–0.69)
**Multi-rater**	0.87	0.99	0.61	0.95	0.74 (0.67–0.82)

*Po, Observed agreement; Max, Maximum; CL, Confidence limits; PABAK, Prevalence-adjusted bias-adjusted Kappa*.

Regarding the intra-rater agreement (Table 2), the observed agreement (Po) was high with values >80% for the four non-expert raters and a value of 0.87 for multiraters^*^experts (Maximum Po = 0.99). The four non-expert raters obtained a Kappa index ≥0.44 (Maximum Kappa ≥0.70) with a value of 0.61 for multiraters^*^experts. The PABAK index ranged between 0.59 and 0.80 for the four non-expert raters with a multiraters^*^experts' value of 0.74 (95% CL = 0.67–0.82), which also implies a “substantial” agreement.

**Figure 2:**
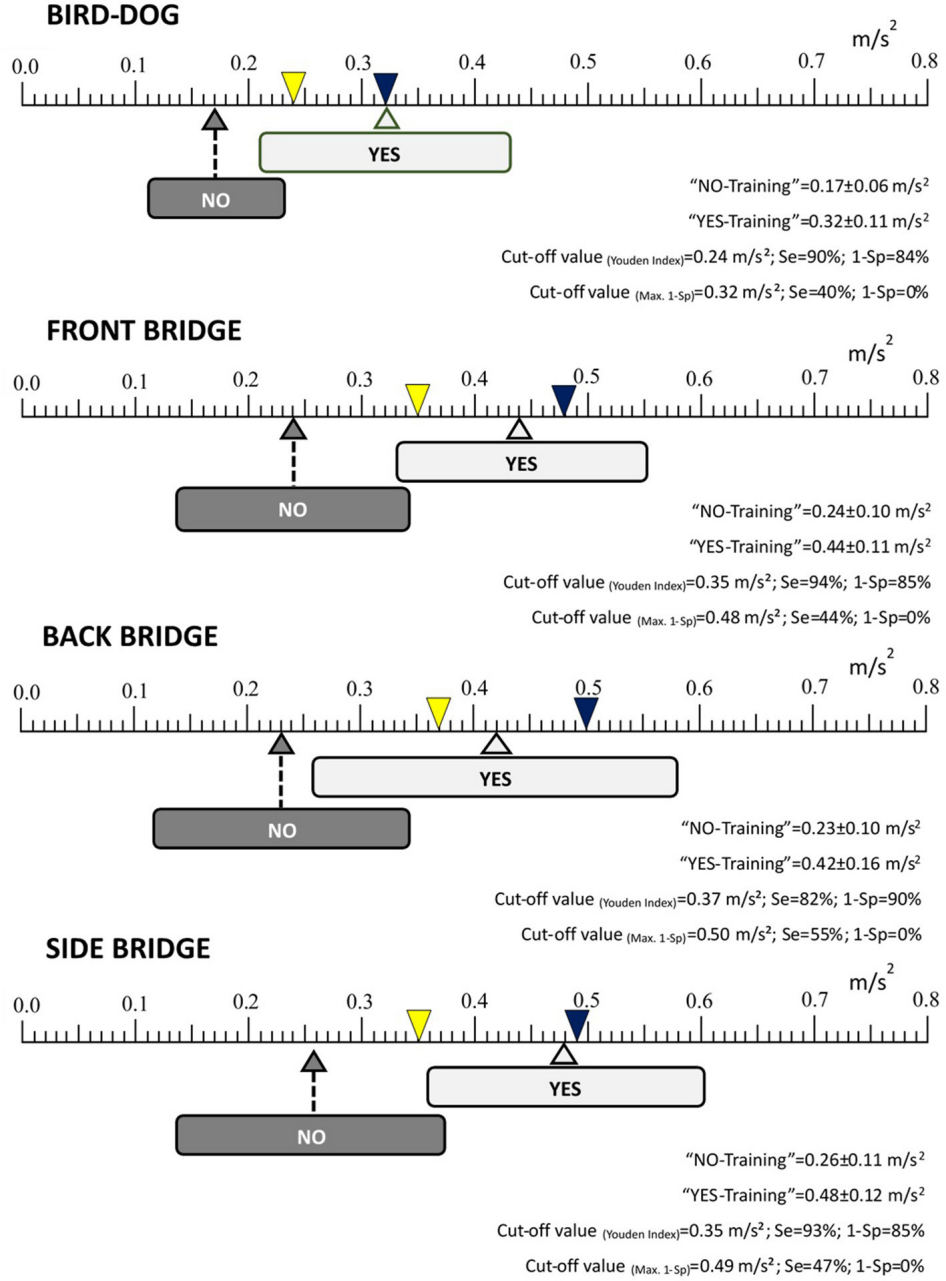
Mean accelerations (±standard deviation) and cut-off values of the core stability exercise variations rated as “YES-Training level” and “NO-Training level”. Se, Sensitivity; 1-Sp, Specificity. Each arrow points (on an acceleration/intensity scale ranging between 0 to 0.8 m/s^2^) the average pelvic acceleration value of all the exercise variations rated by the experts as “YES-Training level” (light gray) or as “NO-Training level” (dark gray). The width of each light gray and dark gray rectangle represents the standard deviation. The yellow inverted triangle indicates the cut point using Youden Index while the blue inverted triangle indicates the cut point minimizing 1-Sp.

**Figure 3:**
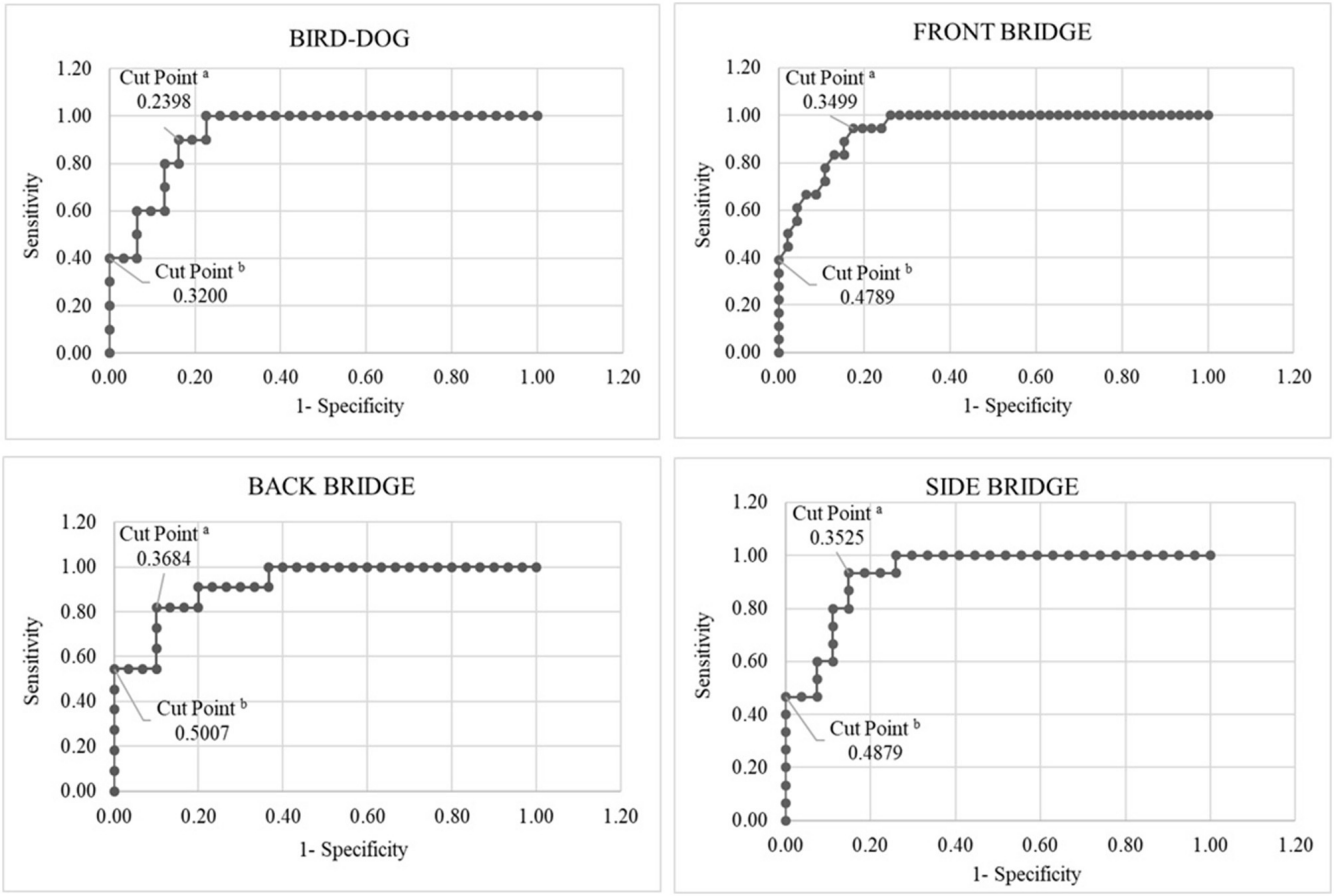
Receiver operating characteristic curves linking the expert ratings with the pelvic accelerations obtained in each of the core stability exercises. ^a^Cut point using Youden Index; ^b^Cut point minimizing 1-Sp.

The ANOVA showed differences between the exercise variations rated as YES-Training level and those rated as NO-Training level for all the CS exercises (*p* ≤ 0.001, with a Hedge's 1.2 < g <2.5 effect size). The mean pelvic accelerations for the CS exercise variations rated as YES-Training level ranged from 0.32 to 0.48 m/s^2^ (Figure 2), while the mean pelvic accelerations for the CS exercises rated as NO-Training level ranged from 0.17 to 0.26 m/s^2^ (Figure 2). Regarding the ROC curve analysis using the Youden Index, the cut-off points for the four CS exercises were (Figures 2, 3): bird-dog = 0.24 m/s^2^ (AUC: 0.923; Sensitivity: 0.90; 1-Sp: 0.16); front bridge = 0.35 m/s^2^ (AUC: 0.946; Se: 0.94; 1-Sp: 0.15); back bridge = 0.37 m/s^2^ (AUC: 0.921; Se: 0.82; 1-Sp: 0.10); and side bridge = 0.35 m/s^2^ (AUC: 0.931; Se: 0.93; 1-Sp: 0.15). In addition, the global cut-off point for the three bridging exercises was 0.35 m/s^2^ (AUC: 0.912; Se: 0.87; 1-Sp: 0.12). On the other hand, the cut-off points for the four CS exercises when minimizing 1-Sp were (Figures 2, 3): bird-dog = 0.32 m/s^2^ (Se: 0.40; 1-Sp: 0.00); front bridge = 0.48 m/s^2^ (Se: 0.44; 1-Sp: 0.00); back bridge = 0.50 m/s^2^ (Se: 0.55; 1-Sp: 0.00); and side bridge = 0.49 m/s^2^ (Se: 0.46; 1-Sp: 0.00). Furthermore, the global cut-off point for the three bridging exercises was 0.50 m/s^2^ (Se: 0.31; 1-Sp: 0.00).

## Discussion

Considering the fact that the lack of training load control, mainly exercise intensity, is one of the main limitations of the CS training programs found in both, the scientific literature and the practical settings, this study: (i) provides new observational screening guidelines to decide if a CS exercise variation represents an adequate training intensity level for a given participant; (ii) analyzes the inter-and intra-rater agreement when using the screening guidelines; and (iii) establishes pelvic acceleration thresholds based on the relationships between expert raters' assessments and pelvic accelerations recorded with a smartphone accelerometer.

To the best of our knowledge this is the first study which has developed screening guidelines to try to establish the intensity of some of the most popular CS exercises. The expert and non-expert raters used these guidelines to select those CS exercise variations which in their opinion represented an intensity level that clearly challenged CS. The inter-rater analysis showed a substantial-high level of agreement between the expert and non-expert raters (PABAK = 0.69; 95% CL = 0.60–0.77), with a high percentage of observed agreement (Po = 84%; Maximum Po = 0.98). This high level of agreement indicates that, with a single training session using the observational screening guidelines, the non-expert raters were able to make similar decisions to those of the expert raters about which exercise variations meant an adequate challenge/intensity level for a given participant. In addition, the data also showed a substantial-high level of intra-rater agreement (Po = 87%; Maximum Po = 0.98; PABAK = 0.74; 95% CL = 0.67–0.82), indicating that, after a period of 6 months, the non-expert raters still retained the rating skills developed in the training session at the beginning of the study.

The high level of inter-and intra-rater agreement and the fact that the observational screening guidelines are few and target different aspects of the CS stability exercise performance, i.e., body alignment and postural sway, lead us to believe that they can be easily applied by sport and health professionals. An important factor in enhancing the inter-and intra-rater agreement were the characteristics of the methodology used in the training session. In this sense, the use of videos of CS exercise progressions with which to practice the selection of the most challenging exercise variations and the feedback from the expert raters on how they assess the participant's performance based on the screening guidelines was very useful. Four videos of a person performing CS exercise progressions with expert raters' feedback on CS exercise performance (Supplementary Material 1) and a table related to these videos with the expert rating for each exercise variation (as YES-Training level or NO-Training level), (Supplementary Material 2) are presented in the Supplementary Material to help those sport and health professionals interested in CS exercise design and prescription to use the criteria properly.

Although the screening guidelines provided in the current study could help people with little experience in CS exercise programs to make reliable assessments about the level of CS exercise intensity/difficulty, the correct performance of these assessments always depends on personal decisions. In order to increase the objectivity of these decisions, the use of smartphone accelerometers placed on the pelvis has been proposed to reliably quantify and control the CS exercise intensity (Barbado et al., 2018). However, as there is no information in the literature to interpret the pelvic accelerations during the CS exercises properly, ROC curves linking the expert ratings with the participants' pelvic accelerations were calculated in this study using two methods. As Figures 2, 3 show, the acceleration cut-off points of the back, side and front bridge exercises (which share analogous characteristics) based on the most conservative method, the Youden Index, were very similar (0.37, 0.35 and 0.35 m/s^2^, respectively), with a global cut-off value of 0.35 m/s^2^ and high values of Se (≥82%) and 1-Sp (≥15%).” On the other hand, the bird-dog exercise showed a lower cut-off point (0.21 m/s^2^) with a 90% of Se and a 16% of 1-Sp. This lower cut-off point could be due to the fact that in the bird-dog exercise variations the pelvis has one or two points of support right below (i.e., the pelvis is supported by the lower limbs), while in the bridging exercises the support points are far from the pelvis, leaving the pelvis suspended in the air and leading to a higher oscillation. In addition, having an arm and a leg elevated during several bird-dog exercise variations might have made the body movements more easily noticeable, which could have influenced the expert's decisions. It must be pointed out that the acceleration thresholds based on the Youden index show high Se and 1-Sp values. Specifically, the high Se values observed for the bridging and the bird-dog exercises (≥82%) means that more than 82% of the selected exercise variations (those rated by the experts as YES-Training level) had a mean pelvic acceleration over the cut-off points. Besides, the high 1-Sp values of the CS exercises (≤ 16%) imply that more than 84% of the exercise variations with a mean pelvic acceleration below the cut-off points were rated by the expert as NO-Training level.

Regarding the cut-off points based on minimizing the 1-Sp index, the acceleration cut-off values shown for the back, side and front bridge exercises were also very similar (0.50, 0.49 and 0.48 m/s^2^, respectively), with a global cut-off value of 0.50 m/s^2^. As occurred when using the Youden Index, the bird-dog exercise showed a lower cut-off point (0.32 m/s^2^). These cut-off values, higher and more restrictive than those obtained using the Youden Index, mean that all the CS exercise variations that were rated as NO-Training level by the expert raters had acceleration values below them. Therefore, CS exercise variations showing acceleration scores above 0.50 m/s^2^ could be considered as proper training stimulus according to the experts' ratings. The problem of choosing cut-off acceleration points minimizing 1-Sp is that the Se is low (0.31 ≤ Se ≤ 0.55), and thus, few exercise variations would be available to be used during a CS exercise program.

Based on the ROC curve results, the pelvic acceleration cut off points mentioned above may represent reference thresholds that could help select adequate training intensity levels for young, healthy and physically active individuals. From the authors' point of view, choosing cut-off acceleration points based on the Youden index or minimizing 1-Sp presents interesting practical implications. Although it has been proven that the conventional bridge and bird-dog variations do not impose high mechanical stress on the lumbar spine (Axler and McGill, 1997; Kavcic et al., 2004), acceleration thresholds based on the Youden index would be recommended when a training stimulus must be applied with the minimum possible level of mechanical stress (i.e., people without experience in CS exercises, with low levels of physical condition, with history of low back pain, etc.). Conversely, acceleration thresholds based on minimizing 1-Sp would be recommended when it is mandatory to ensure that a CS exercise imposes a sufficient training stimulus and the level of mechanical stress tolerance is high (i.e., athletes, people with experience in CS training, etc.).

Although further research is needed to explore the validity of these acceleration thresholds in the current and other populations, the objective data provided by the smartphone accelerometer could be used together with the observational screening guidelines to improve the decision-making process when establishing the intensity of bridging and bird-dog exercises. In relation to this, Supplementary Material 2 shows the pelvic acceleration and the expert rating for each exercise variation presented in the example videos (Supplementary Material 1). Considering the mean acceleration values obtained in each exercise variation and the acceleration thresholds based on the Youden Index established in this study, some exercise variations that were not rated by the experts as YES-Training level (only based on the observational screening guidelines) could have been rated as YES-Training level if they had known the pelvic acceleration values. In this sense, smartphone accelerometry could be especially useful when the raters have doubts rating a CS exercise based on the screening guidelines, especially if they are not expert raters.

The main limitations of this study are the small sample size and the limited generalization of our results as our participants were young and relatively physically fit. Further research should include participants with different ages, spinal conditions, levels of training, etc. Nevertheless, the characteristics of the physical activities carried out by our participants (type, frequency, intensity, volume, etc.) were very heterogeneous, so the interpretation of our results could be applied to young people with different levels of physical fitness. Another limitation of the current study is that each exercise variation lasted only 15 s, so longer durations could have resulted in different acceleration cut-off points, as pelvic accelerations could change due to neuromuscular fatigue. As aforementioned, an exercise duration of 15 s was established because longer durations may have more impact on muscular endurance than on CS. This study also presents a technical limitation related to the generalization of our acceleration results to other devices. In this sense, although it is expected that the biological variations have a far more significant impact on the pelvic acceleration scores than the device noise, it is not clear how using other smartphones (and thus, other accelerometers) could affect the accuracy of the cut-off acceleration thresholds presented in this study. Finally, the acceleration thresholds were established based on the expert ratings rather than on data from experimental studies and therefore they should be interpreted with caution. Future randomized controlled trials should explore the effect of performing CS exercises at different intensity levels based on the acceleration cut-off points established in this study, which will allow to know the usefulness of these acceleration values to induce CS adaptations. In addition, performing CS interventions with different exercise intensities (i.e., pelvic accelerations) in combination with other training variables (e.g., sets, repetitions, exercise durations, etc.) could help to improve the dose-response characterization of the CS exercise programs.

## Conclusions

To our knowledge, this is the first study that has developed observational screening guidelines to establish the intensity of bridging and bird-dog exercises, finding a substantial-high level of intra-and inter-rater agreement when using these criteria. In addition, ROC curves were performed with the aim of linking the CS exercise ratings based on the screening guidelines and the pelvic accelerations recorded with a smartphone accelerometer. The ROC curves showed global acceleration cut-off values which may represent the minimum training intensity levels for these exercises to produce CS adaptations in young physically active individuals, depending on whether a more restrictive (minimizing 1-Sp) or conservative criteria (Youden Index) is used. Therefore, this study provides new observational screening guidelines (targeting body alignment and postural sway while performing CS exercises) and acceleration thresholds based on smartphone accelerometry to facilitate the decision-making process when setting the intensity of bridging and bird-dog exercises in this population.

## Data Availability Statement

The raw data supporting the conclusions of this article will be made available by the authors, without undue reservation.

## Ethics Statement

The studies involving human participants were reviewed and approved by the Miguel Hernández University Office for Research Ethics (DPS.FVG.02.14) according to the Declaration of Helsinki. The patients/participants provided their written informed consent to participate in this study.

## Author Contributions

All authors listed have made a substantial, direct and intellectual contribution to the work, and approved it for publication.

## Funding

This study was made possible by financial support from the *Ministerio de Econom*í*a y Competitividad* (Plan Nacional de I + D + I; Ref: DEP2014-55167-R), Spain.

## Conflict of Interest

The authors declare that the research was conducted in the absence of any commercial or financial relationships that could be construed as a potential conflict of interest.

## Publisher's Note

All claims expressed in this article are solely those of the authors and do not necessarily represent those of their affiliated organizations, or those of the publisher, the editors and the reviewers. Any product that may be evaluated in this article, or claim that may be made by its manufacturer, is not guaranteed or endorsed by the publisher.

## Supplementary Material

Supplementary Table 1 Didactic material of experts' assessment and mean pelvic acceleration for each variation of the core stability exercises showed in the videos presented in the Supplementary Material 1.

Supplementary Video 1 Participant performing a progression of seven bird-dog exercise variations with experts' comments on the participant's performance.

Supplementary Video 2 Participant performing a progression of seven front bridge exercise variations with experts' comments on the participant's performance.

Supplementary Video 3 Participant performing a progression of seven side bridge exercise variations with experts' comments on the participant's performance.

Supplementary Video 4 Participant performing a progression of seven back bridge exercise variations with experts' comments on the participant's performance.
