# Supplementary material for: Observational Screening Guidelines and Smartphone Accelerometer Thresholds to Establish the Intensity of Some of the Most Popular Core Stability Exercises
Source: Front Physiol. 2021 Oct 22;12:751569. doi: 10.3389/fphys.2021.751569 (PMC8570278; doi:10.3389/fphys.2021.751569)
Supplement: Supplementary Table 1 — Didactic material of experts' assessment and mean pelvic acceleration for each variation of the core stability exercises showed in the videos presented in the Supplementary Material 1. [file Table_1.DOCX]

| **Annexed material 2.** Didactic material of experts’ assessment and mean pelvic acceleration for each variation of the core stability exercises showed in the videos presented in the annexed material 1. | | | |
| --- | --- | --- | --- |
| **Exercise variation** | **Pelvic acceleration**  **(m/s^2^)** | **Expert rating** | **Experts’ comments on the participant’s performance** |
| Bird-dog 1 | 0.151 | NO | The participant shows a continuous and light lumbo/pelvic and lower limb movement while maintaining the posture |
| Bird-dog 2 | 0.170 | NO | The participant shows a continuous and light lumbo/pelvic and lower limb movement while maintaining the posture |
| Bird-dog 3 | 0.184 | NO | The participant shows a continuous and light lumbo/pelvic, upper limb and lower limb movement while maintaining the posture |
| Bird-dog 4 | 0.230 | YES | The participant shows a continuous and moderate lumbo/pelvic, upper limb and lower limb movement while maintaining the posture |
| Bird-dog 5 | 0.497 | YES | The participant shows a great difficulty to maintain the body aligned and to limit trunk, upper limb and lower limb movement while maintaining the posture |
| Bird-dog 6 | 0.325 | YES | The participant shows a continuous and moderate lumbo/pelvic movement while drawing squares in the air with the limbs |
| Bird-dog 7 | - | NO | The participant is not able to maintain the required posture while trying to draw squares in the air with the limbs |
| Back Bridge 1 | 0.114 | NO | The participant maintains his body aligned but shows a continuous and light trunk oscillation while maintaining the posture |
| Back Bridge 2 | 0.361 | NO* | The participant occasionally loses and restores the body alignment and shows a continuous and light trunk oscillation while maintaining the posture |
| Back Bridge 3 | 0.160 | NO | The participant maintains the body aligned but continuously shows a light trunk oscillation while maintaining the posture |
| Back Bridge 4 | 0.639 | YES | The participant continuously loses and restores the body alignment and shows a great difficulty to limit trunk movement while maintaining the posture |
| Back Bridge 5 | 0.369 | YES | The participant maintains the body alignment but shows a continuous and moderate trunk oscillation while maintaining the posture |
| Back Bridge 6 | 0.596 | YES | The participant shows a great difficulty to maintain the body alignment and to limit trunk movement while maintaining the posture |
| Back Bridge 7 | - | NO | The participant is not able to maintain the required posture during the whole exercise |
| Front Bridge 1 | 0.126 | NO | The participant maintains his body aligned and restricts trunk movement while maintaining the posture |
| Front Bridge 2 | 0.402 | NO* | The participant maintains his body aligned but occasionally shows a light trunk oscillation while maintaining the posture |
| Front Bridge 3 | 0.466 | YES | The participant maintains the body aligned but often shows a moderate trunk oscillation while maintaining the posture |
| Front Bridge 4 | 0.430 | NO* | The participant maintains his body aligned but shows a continuous and light trunk oscillation while maintaining the posture |
| Front Bridge 5 | 0.580 | YES | The participant occasionally loses and restores the body alignment and often shows a moderate trunk oscillation while maintaining the posture |
| Front Bridge 6 | 0.566 | YES | The participant shows a great difficulty to limit trunk movement, showing a continuous trunk oscillation around the appropriate posture |
| Front Bridge 7 | 0.595 | YES | The participant shows a great difficulty to limit trunk movement, showing a continuous trunk oscillation around the appropriate posture |
| Side Bridge 1 | 0.396 | NO* | The participant maintains the body aligned but shows a continuous and light trunk oscillation while maintaining the posture |
| Side Bridge 2 | 0.475 | YES | The participant occasionally loses and restores the body alignment and often shows a moderate trunk oscillation while maintaining the posture |
| Side Bridge 3 | 0.764 | YES | The participant shows a great difficulty to maintain the body aligned and a high and continuous trunk oscillation while maintaining the posture |
| Side Bridge 4 | 0.566 | YES | The participant loses and restores the body alignment and continuously shows a moderate to high trunk oscillation |
| Side Bridge 5 | 0.578 | YES | The participant shows a great difficulty to maintain the body aligned and a moderate and continuous trunk oscillation while maintaining the posture |
| Side Bridge 6 | - | NO | The participant is not able to maintain the required posture during the whole exercise |
| Side Bridge 7 | - | NO | The participant is not able to maintain the required posture during the whole exercise |
| Mean pelvic acceleration is not presented in those exercise variations in which the participant was not able to maintain the required posture during the exercise.  Expert rating: NO means “NO-Training level” and YES means “YES-Training level”. * exercise variations rated as “NO-Training level” but that could be rated as “YES-Training level” considering the acceleration values.  Note that the person who performed the CS exercises had similar characteristics to the participants in this study: male, age=28 years, height=185 cm, mass=82 kg, physical activity=5 sessions per week of 60 min of moderate cycling exercise. | | | |
